# Genome Microevolution of Chikungunya Viruses Causing the Indian Ocean Outbreak

**DOI:** 10.1371/journal.pmed.0030263

**Published:** 2006-05-23

**Authors:** Isabelle Schuffenecker, Isabelle Iteman, Alain Michault, Séverine Murri, Lionel Frangeul, Marie-Christine Vaney, Rachel Lavenir, Nathalie Pardigon, Jean-Marc Reynes, François Pettinelli, Leon Biscornet, Laure Diancourt, Stéphanie Michel, Stéphane Duquerroy, Ghislaine Guigon, Marie-Pascale Frenkiel, Anne-Claire Bréhin, Nadège Cubito, Philippe Desprès, Frank Kunst, Félix A Rey, Hervé Zeller, Sylvain Brisse

**Affiliations:** 1Centre National de Référence des Arbovirus, Institut Pasteur, Lyon, France; **2**Plate-forme Génotypage des Pathogènes et Santé Publique (PF8), Institut Pasteur, Paris, France; **3**Laboratoire de Microbiologie, Hôpital St Pierre, St Pierre, Ile de la Réunion, France; **4**Plate-forme Intégration et Analyse Génomique, Institut Pasteur, Paris, France; **5**Unité de Virologie Structurale, Institut Pasteur, Paris, France; **6**Centre National de la Recherche Scientifique/Institut National de la Recherche Agronomique UMR 2472/1157, Paris, France; **7**Unité des Interactions Moléculaires Flavivirus-Hôtes, Institut Pasteur, Paris, France; **8**Unité de Virologie, Institut Pasteur, Antananarivo, Madagascar; **9**Laboratoire de Biologie Médicale, Centre Hospitalier de Mayotte, Mamoudzou, Mayotte, France; **10**Disease Surveillance and Sexually Transmitted Infections Unit, Seychelles Public Health Laboratory, Ministry of Health and Social Services, Victoria, Mahe, Seychelles; **11**Université Paris, XI-Orsay, Paris, France; **12**Unité de Génomique des Microorganismes Pathogènes and Centre National de la Recherche Scientifique URA 2171, Institut Pasteur, Paris, France; **13**Centre National de la Recherche Scientifique URA 1930, Paris, France; Washington University School of MedicineUnited States of America

## Abstract

**Background:**

A chikungunya virus outbreak of unprecedented magnitude is currently ongoing in Indian Ocean territories. In Réunion Island, this alphavirus has already infected about one-third of the human population. The main clinical symptom of the disease is a painful and invalidating poly-arthralgia. Besides the arthralgic form, 123 patients with a confirmed chikungunya infection have developed severe clinical signs, i.e., neurological signs or fulminant hepatitis.

**Methods and Findings:**

We report the nearly complete genome sequence of six selected viral isolates (isolated from five sera and one cerebrospinal fluid), along with partial sequences of glycoprotein E1 from a total of 127 patients from Réunion, Seychelles, Mauritius, Madagascar, and Mayotte islands. Our results indicate that the outbreak was initiated by a strain related to East-African isolates, from which viral variants have evolved following a traceable microevolution history. Unique molecular features of the outbreak isolates were identified. Notably, in the region coding for the non-structural proteins, ten amino acid changes were found, four of which were located in alphavirus-conserved positions of nsP2 (which contains helicase, protease, and RNA triphosphatase activities) and of the polymerase nsP4. The sole isolate obtained from the cerebrospinal fluid showed unique changes in nsP1 (T301I), nsP2 (Y642N), and nsP3 (E460 deletion), not obtained from isolates from sera. In the structural proteins region, two noteworthy changes (A226V and D284E) were observed in the membrane fusion glycoprotein E1. Homology 3D modelling allowed mapping of these two changes to regions that are important for membrane fusion and virion assembly. Change E1-A226V was absent in the initial strains but was observed in >90% of subsequent viral sequences from Réunion, denoting evolutionary success possibly due to adaptation to the mosquito vector.

**Conclusions:**

The unique molecular features of the analyzed Indian Ocean isolates of chikungunya virus demonstrate their high evolutionary potential and suggest possible clues for understanding the atypical magnitude and virulence of this outbreak.

## Abstract 2

Viral genome sequence isolated from 6 patients and partial sequences of isolates from 121 patients at different stages and locations of the outbreak reveals unique and evolving genetic features.

## Introduction

Chikungunya virus (CHIKV) is a mosquito-transmitted alphavirus belonging to family Togaviridae [ 1, 2]. It was isolated for the first time from a Tanzanian outbreak in 1952 [ 3]. It is responsible for an acute infection of abrupt onset, characterized by high fever, arthralgia, myalgia, headache, and rash [ 4, 5]. Poly-arthralgia, the typical clinical sign of the disease, is very painful. Symptoms are generally self-limiting and last 1–10 d. However, arthralgia may persist for months or years. In some patients, minor hemorrhagic signs such as epistaxis or gingivorrhagia have also been described.

CHIKV is geographically distributed in Africa, India, and South-East Asia. In Africa, the virus is maintained through a sylvatic transmission cycle between wild primates and mosquitoes such as *Aedes luteocephalus, A. furcifer,* or A. taylori [ 4]. In Asia, CHIKV is transmitted from human to human mainly by A. aegypti and, to a lesser extent, by A. albopictus through an urban transmission cycle. Since the 1952 Tanzania outbreak, CHIKV has caused outbreaks in East Africa (Tanzania and Uganda), in Austral Africa (Zimbabwe and South Africa), in West Africa (Senegal and Nigeria), and in Central Africa (Central African Republic and Democratic Republic of the Congo) [ 4]. The most recent epidemic re-emergence was documented in 1999–2000 in Kinshasa, where an estimated 50,000 persons were infected [ 6]. Since the first documented Asian outbreak in 1958 in Bangkok, Thailand, outbreaks have been documented in Thailand, Cambodia, Vietnam, Laos, Myanmar, Malaysia, Philippines, and Indonesia [ 4, 5]. The most recent epidemic re-emergence was documented in 2001–2003 in Java, after 20 y [ 7]. In both Africa and Asia, the re-emergence was unpredictable, with intervals of 7–8 y to 20 y between consecutive epidemics.

Since the end of 2004, CHIKV has emerged in the islands of the south-western Indian Ocean. Between January and March 2005, more than 5,000 cases were reported in Comoros. Later in 2005, the virus has circulated in the other islands, i.e, Mayotte, Seychelles, Réunion, and Mauritius. Starting in December 2005, the rainy season gave rise to a renewed epidemic circulation of the virus. Since January 1, 2006, several thousands cases were reported in each of Mayotte, Mauritius, and Seychelles islands ( http://www.invs.sante.fr, 21 April, 2006). The most affected island is Réunion (total population: 770,000), with an estimated 244,000 cases (16 April, 2006). More recently, circulation of the virus has also been documented in Madagascar and in India.

In Réunion Island, the first documented cases were patients coming back from Comoros in March 2005. More than 3,000 cases were reported from March to June [ 8]. Transmission was limited (50–100 cases per week) during the winter season of the southern hemisphere, and a major upsurge was observed from mid-December [ 8], with an estimated 12,400 cases in 2005 and an estimated 231,600 cases in 2006 (16 April, 2006). The peak incidence in 2006 was observed during the second week of February, with more than 45,000 cases. The number of cases has now decreased, with an estimated 3,000 cases during the second week of April. Since March 2005, 123 patients with a confirmed CHIKV infection have developed severe clinical signs (neurological signs or fulminant hepatitis) that justified hospitalization in an intensive care unit. Several cases of encephalopathy and major algic syndrome have been associated with vertical transmission of the virus ( http://www.invs.sante.fr).

CHIKV is an enveloped, positive-strand RNA virus. To date, two CHIKV complete nucleotide sequences have been determined, for the strains Ross and S27 [ 9], both isolated from patients during the 1952 Tanzania outbreak. Another complete nucleotide sequence has been determined for a strain isolated in A. furcifer during the Senegal 1983 outbreak (accession no AY726732). Khan et al [ 9] showed that the S27 genome was similar in its structure to that of other alphaviruses and that O'nyong-nyong virus (ONN) was the closest relative to CHIKV. In addition, phylogenetic analyses based on partial E1 sequences from African and Asian isolates revealed the existence of three distinct CHIKV phylogroups: one containing all isolates from West Africa, one containing isolates from Asia, and one corresponding to Eastern, Central, and Southern African isolates [ 10]. Strains isolated in 1999–2000 in the Democratic Republic of Congo belonged to the latter phylogroup [ 6].

In the present study, we determined the nearly complete nucleotide sequences of viruses isolated from six patients originating from Réunion and Seychelles islands. In addition, partial E1 sequences were determined from sera or cerebrospinal fluid (CSF) from a total of 127 patients from Réunion, Seychelles, Madagascar, Mayotte, and Mauritius. Our objectives were to determine the genome structure as well as the unique molecular features of the Indian Ocean outbreak isolates, which may distinguish them from other reported CHIKV and alphavirus sequences. In addition, the phylogenetic origin and the diversity and microevolution of the CHIKV strains responsible for the Indian Ocean outbreak were investigated.

## Methods

### Patients

The 127 patients for whom partial or complete CHIKV nucleotide sequences were determined originated from Réunion ( *n =* 89), Seychelles ( *n =* 3), Madagascar ( *n =* 8), Mayotte ( *n =* 23), and Mauritius ( *n =* 4). Characteristics of the patients and biological samples are listed in Table 1.

**Table 1:**
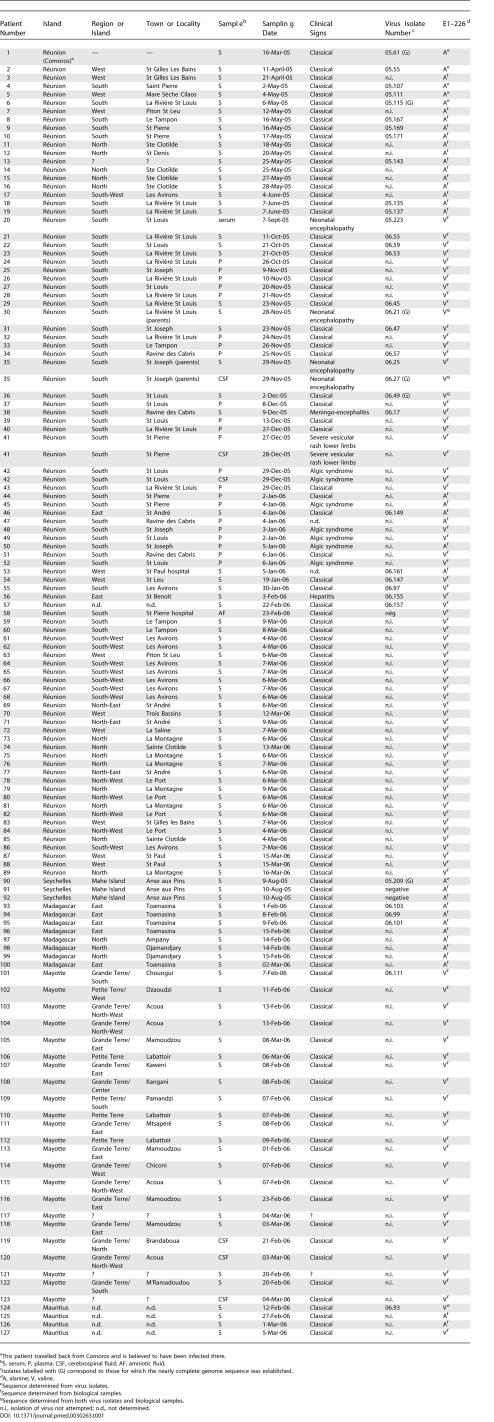
Patient Characteristics

### Specific Mouse Immune Sera and Indirect Immunofluorescence Assay

Mouse hyperimmune ascitic fluid (HMAF) directed against CHIKV was made at the Pasteur Institute. HMAFs against French neurotropic virus strain of yellow fever virus, Hawaï strain of dengue type-1 virus, or IS-98-ST1 strain of West Nile virus were previously described [ 11]. Briefly, specific HMAFs were obtained by repeated immunization of adult mice with crude extracts of virus-infected brains from newborn mice followed by the inoculation of sarcoma 180. Mouse antisera were collected 1 mo after the first immunization. All animal experiments were conducted in accordance with the guidelines of the Office Laboratory of Animal Care at the Pasteur Institute.

For indirect immunofluorescence analysis, mosquito cells were fixed with methanol/acetone (7 Vol/3 Vol) on glass spots at −20 °C for 20 min. Briefly, the fixed cells were incubated with specific HMAFs at a 1:200 dilution in PBS at room temperature for 20 min. After extensive washing with PBS, cells were further incubated with FITC-conjugated goat anti-mouse IgG antibody (Pierce Biotechnology, Rockford, Illinois, United States) at a 1:100 dilution in PBS. The slides were examined using a fluorescence microscope.

### CHIKV Isolation and Viral RNA Extraction

CHIKVs were isolated from either human serum or CSF ( Table 1). A. albopictus C6/36 cells were inoculated with 1 ml of serum or CSF diluted 1:10 in Leibovitz-L15 medium (Invitrogen/Gibco, Carlsbad, California, United States). The cells were grown at 28 °C in Leibovitz-L15 medium supplemented with 5% heat-inactivated foetal bovine serum (FBS) and 10% tryptose-phosphate. Cells and supernatants were harvested after the first passage (5 d) and the second passage (7 d). The virus isolates were identified as CHIKV by indirect immunofluorescence using anti-CHIKV HMAF. In the case of clinical isolates 05.115, 06.21, 06.27, and 06.49, whose genomes were sequenced, absence of yellow fever virus, dengue type-1 virus, and West Nile virus was confirmed by immunofluorescence assay using specific HMAF.

Extraction of viral RNA from the CHIKV isolates was performed using the NucleoSpin RNA II kit (Machery-Nagel, Düren, Germany) or the QIAAmp Viral Minikit (Qiagen, Courtaboeuf Cedex, France) according to manufacturer's recommended procedures. The sequence of the non-structural region of isolates 05.115, 06.21, 06.27, and 06.49 was determined from RNA extracted from supernatants harvested after the second passage. All other CHIKV isolates sequences were obtained using template RNA extracted from the first passage. Extraction of viral RNA from biological specimens was performed using the QIAAmp Viral Minikit.

### Focus Immunoassay

A. pseudoscutellaris AP61 cells were grown in 24-well tissue culture plates in Leibovitz L-15 growth medium with 10% FBS for 24 h. Mosquito cell monolayers were washed once with Leibovitz L-15, and 0.2 ml of Leibovitz L-15/2% FBS was added. Cells were infected with CHIKV in 0.2 ml of Leibovitz L-15/2% FBS and incubated at 28 °C for 1 h. Overlay medium consisting of 0.4 ml of Leibovitz L-15/2% FBS and carboxymethylcellulose (1.6%) was then added, and the tissue culture plates were incubated at 28 °C for 2 d. Foci of infected cells were visualized by focus immunoassay as previously described [ 11]. The cells were washed with PBS, fixed with 3% paraformaldehyde in PBS for 20 min, and permeabilized with 0.5 % Triton X-100 in PBS for 4 min at room temperature. The fixed cells were incubated for 20 min at 37 °C with 1:2,000 dilution of HMAF directed against CHIKV. Horseradish peroxidase-conjugated goat anti-mouse IgG antibody was used as the second antibody (1:100 dilution) at 37 °C for 20 min. Foci were visualized with DAB peroxidase substrate (Sigma, St. Louis, Missouri, United States). Computing of focus sizes was performed with program AxioVision version 4.5 (Carl Zeiss, Oberkochen, Germany) using the automeasure function.

### Nucleotide Sequencing

Primers ( Table S1) were designed based on the nucleotide sequence of the S27 strain. RT-PCR was performed using the Titan One Tube RT-PCR kit (Roche, Meylan, France). RT-PCR fragments were purified by ultrafiltration prior to sequencing (Millipore, Molsheim, France). Sequencing reactions were performed using the BigDye Terminator v1.1 cycle sequencing kit (Applied Biosystems, Foster City, California, United States) and purified by ethanol precipitation. Sequence chromatograms were obtained on automated sequence analysers ABI3100 or ABI3700 (Applied Biosystems). All amplicons were sequenced on both strands.

### Assembly of Genome Sequences and Sequence Analysis

Contig assembly was performed independently by distinct operators and software, using either BioNumerics version 4.5 (Applied-Maths, Sint-Martens-Latem, Belgium) or PhredPhrap/Consed [ 12]. Both analyses yielded exactly the same consensus sequence for all strains. A single contig was obtained for the six isolates. Sequence alignments and computation of substitution tables were performed using programs BioNumerics, DNASP version 4.10 [ 13], and DAMBE version 4.2.13 [ 14]. Alignments of nucleotide and amino acid (aa) sequences against selected alphavirus sequences were performed with ClustalW version 1.7 [ 15]. Sequence identities were computed with the PHYLIP package [ 16]. RNA secondary structure was predicted with the Vienna RNA secondary structure server [ 17]. Neighbour-joining trees were constructed using MEGA version 3.1 [ 18] with the Kimura-2 parameter corrections of multiple substitutions. Reliability of nodes was assessed by bootstrap resampling with 1,000 replicates. Amounts of synonymous substitutions per synonymous site (Ks) and of non synonymous substitutions per non synonymous site (Ka) were estimated using DNASP. RDP2 [ 19] was used to detect putative mosaic sequences.

### 3D Structure Modelling

The crystallographic structure of the ectodomain of the glycoprotein E1 of Semliki Forest virus (SFV) at neutral pH [ 20] was used as a template to model and analyze the two aa mutations of the Indian Ocean isolates. The 3D structure figure was prepared using the program RIBBONS [ 21].

## Results

### Genome Structure and Molecular Signatures of the Indian Ocean Outbreak CHIKVs

#### Genome organization.

We determined the nearly complete genome sequences of six CHIKV isolates (05.115, 05.61, 05.209, 06.21, 06.27, and 06.49) representing distinct geographic origins, time points, and clinical forms ( Table 1) of the Indian Ocean outbreak. 11,601 nucleotides were determined, corresponding to positions 52 (5′ non-translated region [NTR]) to 11,667 (3′NTR, end of third repeat sequence element) in the nucleotide sequence of the 1952 Tanzanian isolate S27 (total length 11,826 nt). There were three insertion/deletion events between S27 and Réunion isolates, two of which were observed in the 3′NTR. First, the internal poly-A stretch of 14 nucleotides observed in S27 (11,440–11,443) and corresponding to a probable internal poly-A site [ 9] was replaced by a stretch of only 5 A in Indian Ocean isolates, similar to what was observed in other CHIKVs, e.g., the Ross strain. Second, one A was missing in Indian Ocean isolates in a 5-A stretch at S27 position 11,625. Finally, one codon was missing in isolate 06.27, corresponding to nsP3 codon 460, at which all other Indian Ocean isolates analyzed and available alphavirus sequences are GAA, coding for Glu.

The genome sequences of the six isolates presented in this paper were similar to those previously reported for alphaviruses [ 9, 22, 23]. Coding sequences consisted of two large open reading frames of 7,422 nt and 3,744 nt encoding the non-structural polyprotein (2,474 aa) and the structural polyprotein (1,248 aa), respectively. The non-structural polyprotein is the precursor of proteins nsP1 (535 aa), nsP2 (798 aa), nsP3 (530 aa), and nsP4 (611 aa), and the structural polyprotein is the precursor of proteins C (261 aa), E3 (64 aa), E2 (423 aa), 6K (61 aa), and E1 (439 aa). Cleavage sites characteristic of the alphavirus family and glycosylation sites in E3, E2, and E1 were conserved. A 65-nt junction sequence was identified between the stop codon (TAG, 7499–7501) of the non-structural open reading frame and the start codon (7567–7569) of the structural open reading frame. The 5′NTR ended at position 76. The 3′NTR region started at position 11,314 and contained three repeat sequence elements with predicted secondary structures ( Figure S1) that were consistent with previous work [ 9].

#### Differences between Indian Ocean outbreak isolates and strain S27.

Compared to strain S27, Réunion isolate 05.115 (the most closely related to S27; see below) showed 28 aa changes (1.13%) in the non-structural proteins ( Table S2), with the highest proportion in nsP3 (2.26%) and the lowest in nsP2 (0.6%). Ten out of 12 aa changes in nsP3 were concentrated between positions 326 and 524 (5.0% variation), similar to findings in ONN viruses [ 24]. One important difference with S27 was that the Indian Ocean isolates exhibited an opal stop codon (UGA) at nsP3 codon 524, instead of Arg (CGA) in S27. This opal codon was observed in related alphaviruses [ 9, 23, 24], and is believed to regulate the expression of nsP4, the putative RNA polymerase, by a read-through mechanism [ 22, 25].

Compared to S27, the structural proteins of isolate 05.115 showed 21 (1.68%) aa substitutions ( Table S3). Notably, envelope protein E2 showed the highest variation, with 14 (3.3%) aa changes, higher than envelope protein E1 (0.68%) and the capsid protein (0.38%). The ratio of rates of evolution of synonymous and non-synonymous sites (Ks/Ka) between S27 and 05.115 isolates was 11.0 for the whole polyprotein, whereas it was only 6.12 for protein E2, probably indicative of a positive selection in favour of aa changes in this immunogenic protein. By comparison, Ks/Ka was 18.75 for the non-structural polyprotein.

#### Indian Ocean outbreak molecular signatures in non-structural proteins and phenotypic variation.

Ten positions (excluding polymorphic positions) had aa that were unique to the non-structural proteins of outbreak isolates, when compared to other CHIKV sequences ( Table 2). Four changes were observed in relatively conserved positions: nsP2–54, nsP2–374, nsP4–254, and nsP4–500. Interestingly, this latter position, which is about 30 aa from the catalytic “GDD” motif, was Leu in the Indian Ocean sequences instead of a Gln in other CHIKV sequences and a strictly conserved Glu in all other alphaviruses. The remaining six changes took place in relatively variable regions ( Table 2).

**Table 2:**
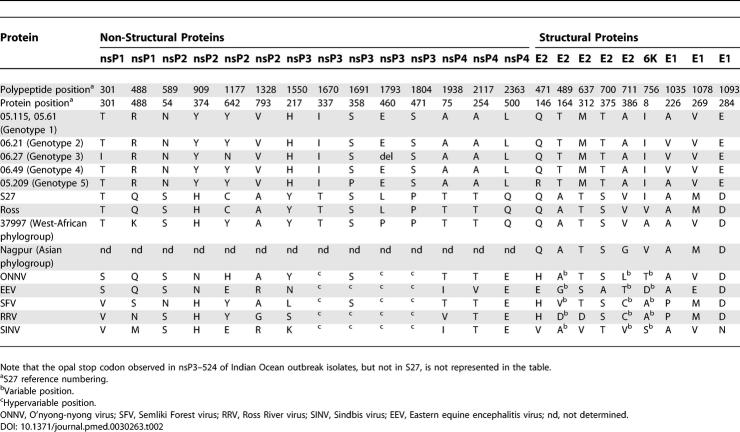
Relevant Amino Acid Changes Identified between Indian Ocean Isolates versus a Selection of Alphavirus Sequences

Additional specific changes were observed in isolates 05.209 (nsP3-S358P) and 06.27 (nsP1-T301I, nsP2-Y642N, and nsP3-460del). Notably, phenotypic assays showed differences for strain 06.27. Focus immunoassay analysis showed that low-passaged CHIKV 05.115, 06.21, 06.27, and 06.49 grown on *A. albopictus* C6/36 (unpublished data) and A. pseudoscuterallis AP61 cells ( Figure S2A) formed mixtures of minute, small, and medium-sized foci. Computing analysis revealed that CHIKV 06.27 exhibited larger foci on AP61 cell monolayers as compared with other strains ( Figure S2B). The particular phenotype of CHIKV 06.27 could be attributed to aa differences in the nsPs, which are involved in viral replication [ 22]. Studies are under way to determine whether nsP1-T301I, nsP2-Y642N, and nsP3-460del changes may alter the growth of CHIKV 06.27 in mosquito cells.

#### Indian Ocean molecular signatures in structural proteins and 3D modelling.

When analyzing the aa sequences of the structural proteins, seven positions (four in E2, one in 6K, and two in E1) were found to be unique to isolates from the Indian Ocean outbreak ( Table 2). Two of these were located in the E2 ectodomain, with Thr 164 and Met 312 being identified in our isolates instead of Ala and Thr, respectively, in all other available CHIKV sequences ( Table 2). The first of these two positions is variable in alphaviruses; it lies in a region previously defined as containing neutralizing epitopes [ 5, 26]. At position 312, Thr is present in other CHIKV, in ONNV, and in SFV, but it varies in other alphaviruses. This position lies in a region identified as important for E1-E2 oligomerization [ 5, 26].

**Figure 1:**
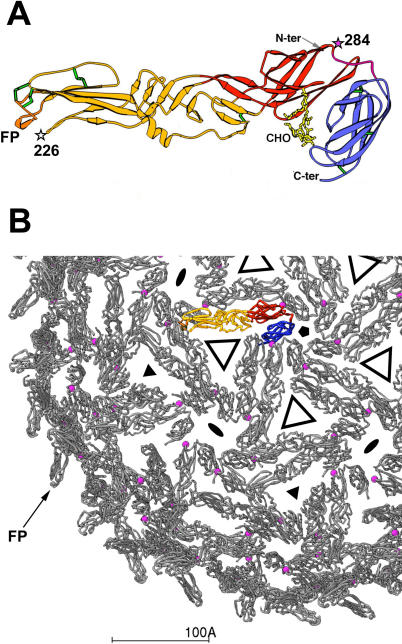
Localization of the E1 Changes on the 3D Structure Modelled from the Crystal Structure of SFV E1 (A) Ribbon diagram of E1, with domain I coloured red, domain II yellow, and domain III blue. Green tubes mark the disulfide bonds. The fusion peptide, at the tip of the molecule (in domain II) is coloured orange and labelled. The N-terminus and the C-terminus observed in the crystal (which is 30 aa upstream of the trans-membrane region) are also labelled. The two unique changes observed in the Indian Ocean isolates are indicated by stars and labelled: positions 226 (white) and 284 (magenta). (B) Partial representation (one octant, slightly extended) of the icosahedral E1 scaffold at the surface of the virion, viewed down a 5-fold symmetry axis. One E1 protomer is highlighted in colours, as in (A); all the others are represented in grey. The location of the some of the icosahedral symmetry axes are drawn as solid black symbols: pentagon for 5-fold axis, triangle for 3-fold axes, ellipse for 2-fold axes (which in the T = 4 lattice of alphaviruses are coincident with quasi 6-fold axes). Open triangles indicate roughly the location of the E2 trimers that interact tightly with E1, covering domain II and the fusion peptide, and presenting the main antigenic sites. The open triangles mark also quasi 3-fold symmetry axes of the T = 4 surface icosahedral lattice. A magenta ball marks the location of Glu 284, at an inter-E1 protomer contact site. This contact is propagated 240 times at the surface lattice (note all pink balls drawn on the grey protomers). Note that the fusion peptide, in orange, is pointing up and away from contacts with other E1 protomers. This is more easily seen at the periphery of the virion, where one of them is labelled (FP). In the virion, this region of E1 is not accessible, covered underneath the E2 molecule [ 20].

In E1, two crucial substitutions were observed: one at residue 284, specific to Indian Ocean isolates, and one at residue 226, present in three out of six Indian Ocean isolates (06.21, 06.27, and 06.49). Both mutations were mapped on the 3D structure (modelled from the crystal structure of SFV E1) in Figure 1. Interestingly, residue 226 was Ala in all previously reported CHIKV sequences ( Table 2), and was also Ala in the Indian Ocean isolates obtained at the beginning of the outbreak (March and May 2005). Subsequent isolates, obtained from patients in November and December 2005, displayed a Val at this position (see below). Although position 226 is relatively variable among alphaviruses, it was observed that a single mutation at this position (Pro to Ser) allowed SFV to adapt to growth in cholesterol-depleted insect cells [ 27, 28].

The other unique aa observed in E1 from Indian Ocean isolates was Glu 284. This is a highly conserved position, which displays an Asp in the majority of alphaviruses or an Asn in SINV ( Table 2). This aa is located at the interface between E1 protomers at the surface of the virion, participating in contacts that make up the icosahedral E1 scaffold ( Figure 1).

### Phylogenetic Analysis

**Figure 2:**
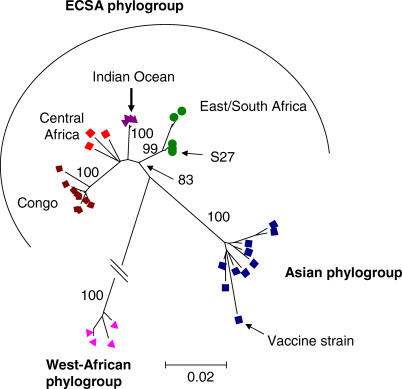
Phylogenetic Relationships among Chikungunya Isolates Based on Partial E1 Nucleotide Sequences Isolates from the Indian Ocean outbreak (Réunion, Seychelles, Mayotte, Mauritius, and Madagascar) represent a distinct clade within a large East-, Central-, and South-African (ECSA) phylogroup. Bootstrap resampling values are indicated at major nodes. The branch leading to West-African phylogroup (of length of approximately 15%) was shortened for convenience. Maximum-likelihood analysis resulted in a very similar phylogeny.

Previous work based on E1 protein sequences showed strong phylogeographic structure of the CHIKV species [ 6, 10]. In order to determine the progenitor phylogroup from which the Indian Ocean outbreak isolates emerged, we compared a 1,044-nt region within the E1 coding sequence (positions 271-1314, i.e., codons 91–438) from the six complete genomes with 29 other available chikungunya sequences ( Table S4). Phylogenetic analysis ( Figure 2) clearly demonstrated that the current Indian Ocean isolates represent a homogeneous clade within a broad group comprising isolates from East, Central, and South Africa (group ECSA, Figure 2). The isolates from the 2000 outbreak in The Democratic of Congo [ 6] also formed a homogeneous clade within group ECSA. There was no ECSA group member showing a significantly closer relationship with the Indian Ocean isolates. Asian isolates were more distantly related to Indian Ocean isolates and constituted the sister group of group ECSA, whereas West-African isolates were even more divergent. Inclusion of other alphaviruses, including the closest relative ONN, placed the root of the chikungunya isolates on the branch leading to the West-African phylogroup (unpublished data).

Comparison of the sequences of Indian Ocean outbreak isolates to the S27 sequence revealed 316 (2.7%) nucleotide substitutions in isolate 05.115 ( Table S5). The Asian clade Nagpur strain showed 5.1% average nucleotide divergence from 05.115, whereas the West-African clade Senegal strain 37997 displayed 15% difference ( Table S5). Interestingly, the latter strain showed complete conservation of an 87-nt portion (9,958–10,045, at the junction between structural proteins 6K and E1) with East-African and Indian Ocean outbreak isolates. Sequence identity in this portion may reflect a past event of genetic recombination between West-African and East-/Central-African strains. Differently, we did not find statistical support ( *p* > 7 × 10 ^−2^) for sequence mosaicism or recombination since the split between S27 and Réunion isolates, although some genomic regions differed in their density of nucleotide polymorphisms.

### Genotypic Variation among Indian Ocean Outbreak Isolates and Microevolutionary Scenario

Specific aa changes in the non-structural proteins were observed in the isolates 05.209 (nsP3-S358P) and 06.27 (nsP1-T301I, nsP2-Y642N, and nsP3-460del). In the structural proteins, change E1-A226V was observed in isolates 06.21, 06.27, and 06.49, and change E2-Q146R in the Seychelles isolate 05.209. In addition to these non-synonymous changes, there were eight silent nucleotide substitutions, observed in 05.209, 06.27, and 06.49 ( Table 3).

**Table 3:**
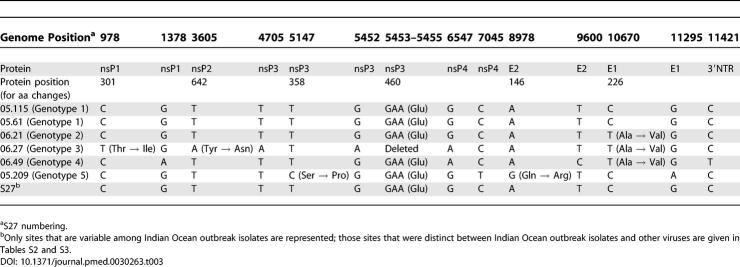
Polymorphisms Observed among Indian Ocean Isolates

**Figure 3:**
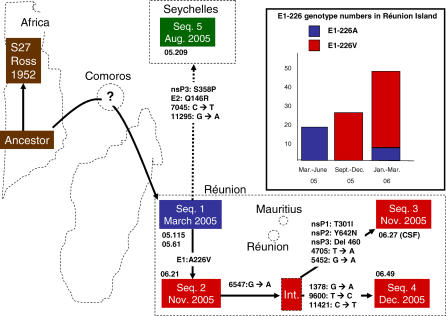
Proposed Evolutionary Scenario of CHIKV Isolates from the Indian Ocean Outbreak The scenario is based on six genome sequences determined by direct sequencing of RT-PCR products obtained using RNA extracts as templates; the sequences (Seq.) thus correspond to consensus sequences of the possible mixture of coexisting genomes (quasispecies). Inset: number of cases of E1-226A and E1-226V at different time intervals in Réunion Island, based on partial E1 sequences. E1-226V was observed in consensus sequences 2, 3, and 4, and therefore most E1-226V isolates genotyped based on partial E1 sequences are likely related to these genotypes. However, the independent appearance of E1-226V in other genotypes cannot be excluded. The location, size, and relative position of the islands and the African border are indicative. Consensus sequence 1 was obtained from a Réunion patient who travelled back from Comoros in March 2005, and from a Réunion Island patient. Sequences 2–4 were sampled in Réunion Island; sequence 5 was sampled in the Seychelles. The strain codes are given under the corresponding sequences. The question mark in Comoros Archipelago indicates that Sequence 1 possibly originates from Comoros. The dotted link between Sequence 1 and Sequence 5 indicates that the transmission route is hypothetical. CSF, cerebrospinal fluid; Int., deduced intermediate sequence, which was not sampled.

A history of probable sequence evolution that occurred during the outbreak ( Figure 3) was deduced from the 14 nucleotide variations observed among the six complete genomes ( Table 3). Isolate 05.61 was initially selected for genome analysis because it was isolated in March 2005, at the onset of the outbreak, from a Réunion patient returning from Comoros Island, where the outbreak had been going on since January 2005. Remarkably, the isolates 05.61 and 05.115 (which was the second earliest isolate analyzed), the African isolate S27, and previous unrelated chikungunya isolates from Africa and Asia were identical at all 14 polymorphic sites. Therefore, the consensus sequence of isolates 05.61 and 05.115 (consensus sequence 1) likely represents the ancestral genotype of the Réunion outbreak. Distribution of the 14 polymorphisms suggested that this founder gave rise to three consensus sequences that likely evolved in four steps. First, substitution at genome position 10,670 (causing the E1 A226V change) gave rise to consensus sequence 2, represented by the late-November 2005 isolate 06.21. Second, a G-to-A synonymous substitution at position 6,547 (nsP4) led to an intermediate sequence, which itself gave rise to two late sequences: consensus sequence 3 (isolate 06.27), following four additional substitutions and one codon deletion ( Table 3), and consensus sequence 4 (06.49), which arose after three distinct synonymous substitutions ( Table 3). A fifth consensus sequence was represented by the Seychelles isolate 05.209 alone, which exhibited four substitutions (two of them causing aa changes in nsP3-S358P and in E2-Q146R) compared to consensus sequence 1 ( Figure 3).

Since Réunion isolates had E1-226A at the beginning of the outbreak and E1-226V later in the epidemics, we compared residue 226 in a total of 92 sequences (from 87 sera, four CSF, and one amniotic fluid) from 89 patients from Réunion Island. Remarkably, the nature of E1–226 differed totally before and after the winter season. Nineteen sequences from patients sampled from 16 March to 7 June, 2005 (including the sequence originating from a traveller back from Comoros) had E1-226A. Between 7 September and end of December 2005, 27 sequences showed E1-226V. Among 46 Réunion sequences from 2006, E1-226V was observed 40 times (87%) and E1-226A was observed six times ( Table 1). In Madagascar and Seychelles sequences, for which the samples were collected when the first clinical cases were suspected (i.e., probably at the beginning of the outbreaks), only the E1-226A was observed. On Mayotte, where the outbreak started in early 2005, only E1-226V was observed in 23 sequences obtained from patients in 2006. In sequences from patients infected in Mauritius, where the outbreak also started in 2005, both E1-226A and E1-226V were observed.

## Discussion

CHIKV is currently causing one of the largest chikungunya fever outbreaks reported in the past 40 y [ 4– 6, 29]. The magnitude of the epidemics has surprised the population, policy makers, and public health specialists, although chikungunya is not uncommon in nearby regions of this part of the world. Our phylogenetic analyses based on partial glycoprotein E1 sequences indicate that the Indian Ocean outbreak was caused by the same strain on Réunion, Seychelles, Mayotte, Madagascar, and Mauritius islands, and show that the outbreak strain is related to East-, Central-, and South-African isolates. Although, to our knowledge, no outbreak was reported recently in East Africa, this scenario is compatible with the human population exchanges between East Africa and Comoros, where the outbreak is believed to have started. Whether the epidemic strain results from the evolution of an enzootic strain, as previously described for eastern equine encephalitis virus [ 30], cannot be excluded. Sequencing of additional isolates from recent outbreaks in Africa and from possible local reservoirs should define more precisely the origin of the Indian Ocean outbreak.

This study represents the first survey, to our knowledge, of intra-outbreak analysis of CHIKV nucleotide variation on a genomic scale. The availability of multiple nearly complete genome sequences allowed us to deduce the probable history of successive molecular evolutionary changes that may have occurred while the outbreak was still ongoing. This evolutionary scenario is the most likely based on the six consensus sequences obtained, although additional isolates and determination of quasispecies heterogeneity are needed to obtain a more precise picture of viral evolution during the outbreak.

Whereas E1-226A was the only genotype observed during the first period of the Réunion outbreak (March–June 2005), our data show the emergence and predominance of genotype E1-226V, which was observed from the beginning of September 2005 and experienced a spectacular rise in frequency. The appearance of E1-226V preceded by at least 3 mo the explosive epidemic peak of mid-December 2005, and the link between this aa change and the rate of transmission thus deserves further investigation. Interestingly, a mutation at residue 226 in SFV was observed to release the cholesterol dependence of the virus [ 27, 28]. It is thus possible that such a mutation provides a selective advantage to the virus in mosquitoes, which are cholesterol auxotrophs. Indeed, in the SFV E1 crystal structure, position 226 is located in the ij loop, in contact with the fusion peptide, in a region of the protein that is predicted to interact with the target membrane. On the other hand, this change may be selectively neutral, and other evolutionary factors such as genetic drift or a founder effect could have favoured E1-226V by chance alone. In Sindbis virus, a change at position 226 alone was not sufficient to release cholesterol dependence [ 31]. Our data thus point to interesting experiments comparing the cholesterol dependence of the isolates differing at position 226 in E1.

To date, only CHIKV laboratory strains, passaged many times on mosquito or mammalian cells, had been entirely sequenced [ 9]. We provide for the first time nearly complete nucleotide sequences of six clinical isolates passaged in vitro only once or twice (see Methods). Limiting the number of passages is crucial because the infecting viral population may correspond to a quasispecies [ 32– 34], i.e., a mixed viral population with genotypes co-existing in an equilibrium governed by a balance between mutation and natural selection. Repeated in vitro passages may act as a filter on this population. For example, the presence in S27 of an Arg codon instead of the opal stop codon in Indian Ocean isolates is probably explained by numerous in vitro passages of S27, as evolution of opal to Arg was observed experimentally in ONN viruses [ 24]. Whereas it may be advantageous for viral quasispecies to maintain the opal codon in vivo, an Arg codon may confer a selective advantage in vitro, as observed for the closely related SFV [ 35]. In the present study, careful inspection of the chromatograms traces identified three codons with double peaks observed both on forward and reverse traces ( Figure S3). In all three cases, alternative bases corresponded to non-synonymous codons that may thus alter viral fitness. It is tempting to speculate that quasispecies diversity in vivo might facilitate the access to different body sites, such as the central nervous system. For example, selection for a subset of genotypes harbouring the changes observed in CSF isolate 06.27 may be associated with invasion of the CSF [ 34]. These observations underscore that the genome sequence of laboratory “reference” strains may not accurately reflect the natural situation, as the genotypic complexity of quasispecies in vivo is subject to erosion by in vitro selection. Since the Indian Ocean isolates sequenced here were subjected to in vitro selection for only a few generations, they probably correspond more closely to the in vivo genotypes than previously sequenced chikungunya strains.

The aa differences detected among the outbreak isolates may relate to biological or pathogenic characteristics of the virus. Although our viral culture results are preliminary, they clearly show phenotypic differences between the unique isolate from CSF (06.27), isolated from a neonatal encephalopathy case, and three other isolates from sera associated with either the classical form of the disease or encephalopathy. The larger foci observed in culture with 06.27 could reflect a higher replication rate of the virus and be linked to the specific aa changes identified in nsP1, nsP2, and nsP3. Single aa changes in nsP1, including a Thr/Ile change (residue 538 of Sindbis virus) [ 36, 37] and a 18-nt deletion in nsP3 have previously been shown to affect neurovirulence in other alphaviruses [ 36– 38]. However, in the absence of nsP1, nsP2, and nsP3 structural data, it is difficult to predict whether the specific aa changes observed in isolate 06.27 can have a structural or functional impact. We also noted that all the viral sequences determined from either the serum or the isolates from three neonatal encephalopathy cases and an adult meningo-encephalitis case had E1-226V. However, as this genotype is also observed in classical forms of the disease, one cannot conclude on a potential link of E1-226V with neuropathogenesis. Host factors also have to be considered in the occurrence of neurological forms of the disease. For example, the blood–brain crossing may be favoured by age or hypertension.

Molecular signatures of the Indian Ocean outbreak genomes were identified when they were compared to all other reported alphavirus sequences. However, these comparisons have to be considered with caution because of a potential sampling bias due to the small number of previous alphavirus sequences. Nevertheless, these features represent interesting targets for future functional studies, as well as for epidemiological follow-up. One particularly interesting feature was the E1-226V residue (see above). Another interesting molecular signature was E1–284 Asp. Although the pseudo-atomic model of the scaffold used is of modest resolution (the resolution of the crystal structure is limited—approaching 3Å—and the model results of fitting this structure into a 9Å resolution cryo-electron microscopy reconstruction), it appears that the side-chain of Asp 284 interacts with the main chain of an adjacent E1 polypeptide in the virion. Indeed, it is in a position compatible with acceptance of a hydrogen bond from a main chain amide in the contacting E1 protomer. Because the packing is very tight (see Figure 1B), it is possible that the longer glutamic acid side chain (which has an extra CH _2_ group compared to Asp or Asn) may introduce a slight distortion at the contact sites, an effect that is propagated by the icosahedral T = 4 symmetry of the virion. Thus, a cooperative effect due to this change at position Asp 284 may play a role in either allowing a less efficient assembly of new particles in infected cells, or a more efficient particle disassembly process during invasion of a new cell, or a combination of both. This information can guide new site-directed mutagenesis studies, using reverse genetics, to test the effect of the Asp/Glu replacement on the virus cycle.

The magnitude and high political profile of the outbreak has underlined the critical lack of knowledge on the biology of CHIKV, contrasting with related model alphaviruses such as Sindbis, Semliki Forest, and Ross River. This situation reflects the fact that chikungunya infection, despite infecting millions of people since its discovery, has been neglected. However, chikungunya disease is clearly responsible for disabling and persistent arthralgia, although it remains unresolved whether the symptoms are due to persistence of the virus or inappropriate immune response [ 39, 40]. In addition, during the Indian Ocean CHIKV outbreak, a small proportion of the patients (about 123 out of 244,000 infected) developed severe clinical signs such as neurological signs or hepatitis. Whereas neurovirulence and neuroinvasiveness are established for several alphaviruses such as eastern equine encephalitis virus and Venezuelan equine encephalitis virus, only two CHIKV strains had previously been isolated from children with clinical signs suggestive of encephalitis and meningitis [ 41, 42]. Whether CHIKV Indian Ocean strains have acquired a higher neurovirulence or neuroinvasiveness certainly deserves investigation.

In the absence of efficient vaccine or antiviral therapy, vector control is at present the only way to limit chikungunya transmission. However, the broad geographic distribution of the mosquito vectors A. albopictus and A. aegyptii [ 43] may allow the expansion of CHIKV to new areas, such as the European or American continents. The molecular data reported here on clinical isolates from the current epidemics should contribute to bridge the gap of knowledge concerning this human pathogen, and will help to provide, in the middle term, more specific and powerful tools to combat it.

## Supporting Information

Alternative Language Abstract S1 Translation of the Abstract into French by IS (28 KB DOC)

Figure S1 Repeat Sequence Elements found in the 3′NTR Region (A) Alignment of repeat sequence elements found in the 3′NTR region of CHIKV genome. All sequences form conserved and stable stem-loop structures in which the less conserved nucleotides around position 20 constitute the loop. Three repeat sequence elements are found in all chikungunya genomes. The first one (RSE1) is inserted before the internal poly-A sequence of S27 genome [ 9], whereas the two others are found downstream of this motif. (B) Predicted secondary structure for RSE1 of isolate 05–115.
(89 KB PPT)

Figure S2 Focus Size Phenotype of CHIKV on Mosquito Cells Mosquito AP61 cells grown in 24-well plates (10 ^5^ cells/well) were infected with 5–10 FFU/well or 50–100 FFU/well of low-passaged CHIKV 05.115, 06.21, 06.27, and 06.49. Infected cells were overlaid with carboxymethylcellulose in Leibovitz L-15 growth medium with 2% FBS for 2 d to allow focus development at 28 °C. Infected cells were fixed with 3% paraformaldehyde in PBS, permeabilized with Triton X-100 in PBS, and foci of CHIK virus replication were immunostained with anti-CHIKV HMAF (dilution 1:2,000) and peroxidase-conjugated goat anti-mouse Ig (dilution 1:100). The histograms depict the total area of viral foci as determined by squared pixels (relative values).
(2.5 MB PPT)

Figure S3 Chromatogram Traces at Three Nucleotide Positions That Showed Mixed Peaks Observed on Both Strands Such peaks can be explained by the existence of quasispecies, i.e., the coexistence of several distinct genotypes in vivo. Position 978 (nsP1–301) showed a C/T peak (Thr/Ile replacement), in three strains (05.115, 06.21, and 06.49), all three sequenced from passage 2 RNA extracts. Interestingly, this site corresponded to the T substitution in Réunion isolate 06.27 (CSF sample, passage 1) instead of a C in isolate 05.61 and in other CHIKV sequences. Position 1016 showed a mixed A/G peak in strain 06.49, which corresponds to either Met or Val at residue nsP1–313. Finally, position 1070 showed an A/G peak in isolates 05.115 and 06.21, which results in Thr or Ala at residue nsP1–331.
(80 KB PPT)

Table S1 Primers Used for RT-PCR and Sequencing (20 KB XLS)

Table S2 Amino Acid Changes Observed between Strain S27 and Indian Ocean Outbreak Strains in the Non-Structural Proteins (21 KB XLS)

Table S3 Amino Acid Changes Observed in the Structural Genes among Strains S27, Ross, and from the Indian Ocean Outbreak (19 KB XLS)

Table S4 Sequences Used for the Phylogenetic Analysis of Partial E1 Sequences (14 KB XLS)

Table S5 Sequence Percent Similarity Based on Amino Acids and Nucleotides (in Parentheses) for the Structural (SP) and Non-Structural (NSP) Proteins of Selected Alphaviruses (17 KB XLS)

### Accession Numbers

The sequences discussed in this paper were deposited in GenBank/EMBL ( http://www.ncbi.nlm.nih.gov/Genbank) databases under the accession numbers AM258990–AM258995. The accession numbers for CHIKV complete nucleotide sequences discussed in this paper are AF490259 for the Ross strain, AF369024 for the S27 strain, and AY726732 for the A. furcifer strain. The Protein Data Bank ( http://www.rcsb.org/pdb) accession number for Semliki Forest Virus E1 protein is 2ALA.

## Abbreviations

aa: amino acid
CHIKV: chikungunya virus
CSF: cerebrospinal fluid
FBS: foetal bovine serum
HMAF: hyperimmune ascitic fluid
ONN: O'nyong-nyong virus
SFV: Semliki Forest virus
