# Supplementary material for: Genome Microevolution of Chikungunya Viruses Causing the Indian Ocean Outbreak
Source: PLoS Med. 2006 May 23;3(7):e263. doi: 10.1371/journal.pmed.0030263 (PMC1463904; doi:10.1371/journal.pmed.0030263)
Supplement: Supplementary file 1 [file pmed.0030263.sd001.doc]

**Supplemetary summar in: French**

**Introduction**

Une épidémie à virus Chikungunya d’une ampleur exceptionnelle sévit actuellement dans les îles du Sud-Ouest de l’Océan Indien. Le virus Chikungunya a, à ce jour, infecté le tiers de la population Réunionnaise. Le signe clinique caractéristique de l’infection est une poly-arthralgie douloureuse et invalidante. En plus des formes arthralgiques, 123 patients avec infection à Chikungunya documentée ont développé des signes cliniques sévères, i.e méningo-encéphalites, encéphalopathies ou hépatite fulminante.

**Méthodes et résultats**

Nous décrivons la séquence quasi-complète du génome de six isolats viraux (issus de 5 sérums et 1 liquide cephalo-rachidien – LCR de patients), ainsi que des séquences partielles (1044 pb) de la glycoprotéine E1 de 127 patients infectés à la Réunion, aux Seychelles, à Madagascar, à Mayotte et à l’île Maurice. Nos résultats indiquent que l’épidémie a pour origine une souche génétiquement proche de souches virales précédemment isolées lors d’épidémies en Afrique de l’Est. Cette souche aurait ensuite évolué selon un schéma évolutif qu’il est possible de retracer. Des signatures moléculaires spécifiques aux isolats de l’Océan Indien ont pu être caractérisées. En particulier, au sein de la séquence des protéines non-structurales, 10 changements d’acides aminés ont été mis en évidence par comparaison avec les séquences déjà publiées. Parmi ces 10 changements, 4 sont localisées dans des régions conservées des protéines nsP2 (contenant les domaines hélicase, protéase et tri-phosphatase) et nsP4 (polymérase du virus). L’isolat obtenu à partir d’un LCR montre 3 substitutions, non retrouvées sur les isolats viraux obtenus à partir de sérums, dans les protéines nsP1 (T301I), nsP2 (Y642N) et nsP3 (délétion de l’acide aminé E460). Deux substitutions notables (A226V et D284E) ont également été mises en évidence dans la glycoprotéine E1. Une modélisation de la structure tri-dimensionnelle de la glycoprotéine E1 nous a permis de localiser ces mutations sur des régions importantes pour le processus de fusion et l’assemblage viral. Chez les patients Réunionnais, la substitution A226V etait absente au de début d’épidémie (mars-juin 2005) mais est observée dans plus de 90% des séquences issues de patients prélevés après septembre 2005, suggérant un succès évolutif possiblement lié à une meilleure adaptation du virus au moustique vecteur.

**Conclusions**

Les caractéristiques moléculaires propres aux isolats de virus Chikungunya de l’Océan Indien démontrent leur haut potentiel évolutif et suggèrent des pistes pour mieux comprendre l’amplitude exceptionnelle et les formes cliniques sévères de cette épidémie.
